# Improving diagnosis accuracy with an intelligent image retrieval system for lung pathologies detection: a features extractor approach

**DOI:** 10.1038/s41598-023-42366-w

**Published:** 2023-10-03

**Authors:** Abdelbaki Souid, Najah Alsubaie, Ben Othman Soufiene, Mohammed S. Alqahtani, Mohamed Abbas, Layal K. Jambi, Hedi Sakli

**Affiliations:** 1 MACS Research Laboratory RL16ES22, National Engineering School of Gabes, Gabes, Tunisia; 2 Department of Computer Sciences, College of Computer and Information Sciences, Princess Nourah bint Abdulrahman University, P.O. Box 84428, 11671 Riyadh, Saudi Arabia; 3 PRINCE Laboratory Research, ISITcom, University of Sousse, Hammam Sousse, Tunisia; 4 Radiological Sciences Department, College of Applied Medical Sciences, King Khalid University, 61421 Abha, Saudi Arabia; 5 BioImaging Unit, Space Research Centre, Michael Atiyah Building, University of Leicester, Leicester, LE17RH UK; 6 Electrical Engineering Department, College of Engineering, King Khalid University, 61421 Abha, Saudi Arabia; 7 Radiological Sciences Department, College of Applied Medical Sciences, King Saud University, P.O. Box 10219, 11433 Riyadh, Saudi Arabia; 8 EITA Consulting, 5 Rue Du Chant Des Oiseaux, 78360 Montesson, France

**Keywords:** Computational biology and bioinformatics, Diseases, Health care

## Abstract

Detecting lung pathologies is critical for precise medical diagnosis. In the realm of diagnostic methods, various approaches, including imaging tests, physical examinations, and laboratory tests, contribute to this process. Of particular note, imaging techniques like X-rays, CT scans, and MRI scans play a pivotal role in identifying lung pathologies with their non-invasive insights. Deep learning, a subset of artificial intelligence, holds significant promise in revolutionizing the detection and diagnosis of lung pathologies. By leveraging expansive datasets, deep learning algorithms autonomously discern intricate patterns and features within medical images, such as chest X-rays and CT scans. These algorithms exhibit an exceptional capacity to recognize subtle markers indicative of lung diseases. Yet, while their potential is evident, inherent limitations persist. The demand for abundant labeled data during training and the susceptibility to data biases challenge their accuracy. To address these formidable challenges, this research introduces a tailored computer-assisted system designed for the automatic retrieval of annotated medical images that share similar content. At its core lies an intelligent deep learning-based features extractor, adept at simplifying the retrieval of analogous images from an extensive chest radiograph database. The crux of our innovation rests upon the fusion of YOLOv5 and EfficientNet within the features extractor module. This strategic fusion synergizes YOLOv5's rapid and efficient object detection capabilities with EfficientNet's proficiency in combating noisy predictions. The result is a distinctive amalgamation that redefines the efficiency and accuracy of features extraction. Through rigorous experimentation conducted on an extensive and diverse dataset, our proposed solution decisively surpasses conventional methodologies. The model's achievement of a mean average precision of 0.488 with a threshold of 0.9 stands as a testament to its effectiveness, overshadowing the results of YOLOv5 + ResNet and EfficientDet, which achieved 0.234 and 0.257 respectively. Furthermore, our model demonstrates a marked precision improvement, attaining a value of 0.864 across all pathologies—a noteworthy leap of approximately 0.352 compared to YOLOv5 + ResNet and EfficientDet. This research presents a significant stride toward enhancing radiologists' workflow efficiency, offering a refined and proficient tool for retrieving analogous annotated medical images.

## Introduction

Lung pathologies encompass a wide range of conditions that can affect the respiratory system, ranging from minor ailments to life-threatening diseases. These conditions can be caused by a variety of risk factors, including exposure to environmental pollutants, infections, genetic predisposition, lifestyle choices, and other underlying health conditions. Infections such as tuberculosis and pneumonia can also increase the risk of developing lung pathologies. These infections can lead to inflammation, scarring, and other damage to the lungs, making individuals more susceptible to developing lung diseases such as bronchiectasis and pulmonary fibrosis. Lung pathologies can have a variety of causes, and the risk of developing these conditions can be influenced by a combination of environmental, genetic, and lifestyle factors. Reducing exposure to environmental pollutants, adopting healthy lifestyle habits, and receiving appropriate medical care for underlying health conditions can help to minimize the risk of developing lung pathologies. Chest X-ray is a commonly used diagnostic tool for detecting lung pathologies. The image produced by chest X-ray provides detailed information about the structure and condition of the lungs, allowing healthcare professionals to identify any abnormalities or changes that may indicate a lung pathology.

**Figure 1:**
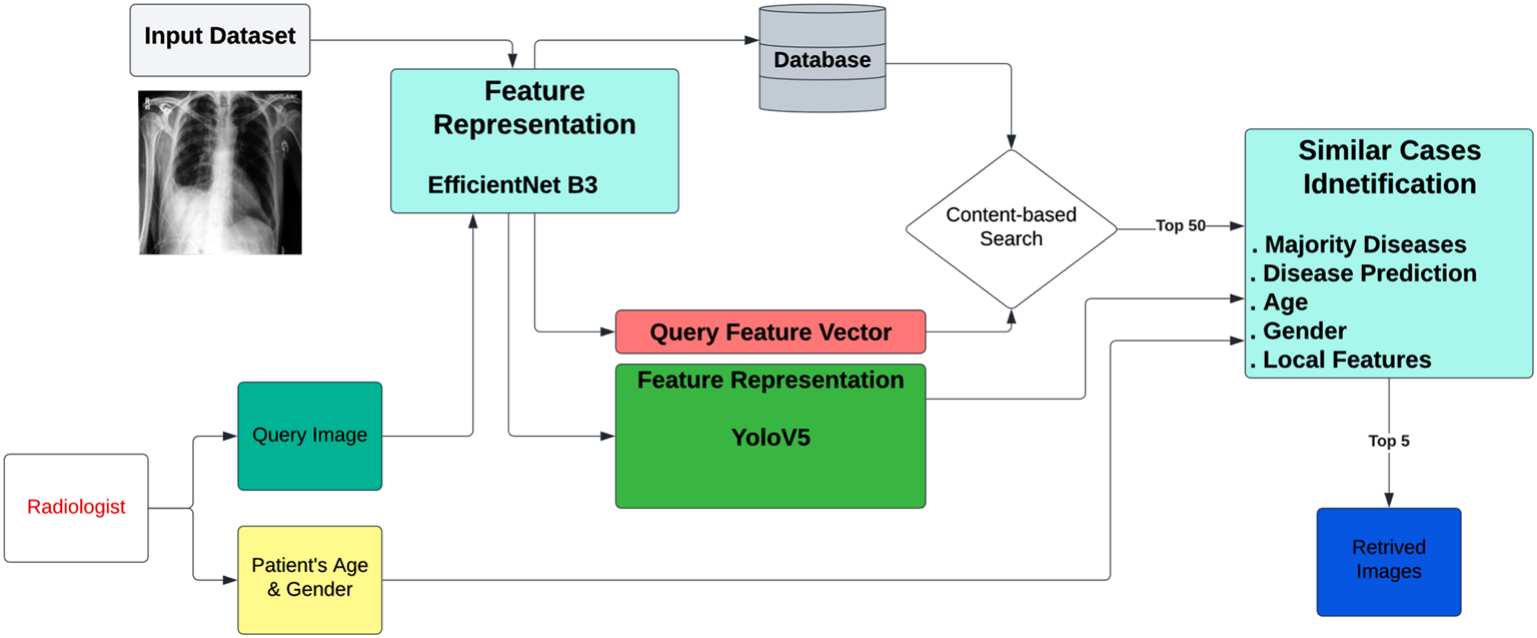
Overview of the CBMIR Architecture, the two main tasks are features representation and features retrieving task.

Access to medical imaging technology has expanded significantly over the last decade, increasing the number of images that radiologists must interpret in their daily workflow ^1^. The effective available time per diagnostic has been diminishing as the ratio of diagnostic demand to the number of radiologists has increased, and this has become a crucial issue when a diagnostic must be backed by confirmatory evidence of a probable suspected diagnosis. In the realm of medical imaging, radiologists often face the challenge of making a diagnosis when confronted with a suspected condition. In such cases, they typically resort to manually searching through public or internal image databases for similar images that can aid in their decision-making process. This manual method is not only time-consuming but also often requires multiple iterations to find the right matching image. Considering the importance of accurate and efficient diagnoses in medical imaging, it is of great significance to investigate the development of disease-targeted content-based image retrieval (CBIR) systems. Such systems would automatically present similar images that match the one being analyzed, thus reducing the time and effort required for manual searching. In light of these considerations, this presents a compelling opportunity for academic research aimed at advancing the development of CBIR systems for medical imaging. By leveraging state-of-the-art techniques in image analysis and deep learning, such systems have the potential to provide radiologists with more efficient and effective tools for making accurate diagnoses, thus significantly advancing the field of medical imaging. CBIR systems' two core functions, features representation and features indexing and search, are crucial components for the effective and efficient retrieval of similar images. In features representation, the goal is to find a compact and descriptive representation of the image that accurately characterizes its content. On the other hand, features indexing, and search focuses on improving the speed and efficiency of the retrieval process. Together, these two tasks form the foundation of CBIR systems, and their effective implementation is critical for their success. Figure 1 illustrates the workflow of a CBIR system. Several studies have been conducted on CBIR. They can be broadly classified into two categories: (1) studies that focus on feature extraction and (2) studies that focus on similarity measures and retrieval algorithms. studies that focus on similarity measures and retrieval algorithms. One such system is an interpretability-driven and attention-driven medical image retrieval system proposed by Wilson Silva et al ^2^. Similarly, IEEE introduces a CBMIR system that employs Stacked Autoencoders for recognizing disease characteristics in medical images ^3^. Numerous CBMIR systems have also emerged, including a comprehensive community-based approach for large-scale content-based X-ray image retrieval pioneered by Nandinee Fariah Haq et al ^4^.

This work contributes to the advancement of medical imaging by introducing a deep learning-based detection model. The goal is to enhance the accuracy of diagnosing lung and thoracic-related pathologies while streamlining the process of examining chest radiographs. Our approach primarily focuses on the task of feature representation, which involves capturing key information from radiographic images. We propose a unique method that combines YOLOv5 and EfficientNet, well-established techniques in image analysis, to create a robust feature extractor.

Our research demonstrates that our approach outperforms conventional methods, as evidenced by higher mean average precision and precision metrics across various pathologies. This advancement signifies progress in intelligent image retrieval systems for medical imaging. By improving accuracy, we aim to reduce the occurrence of false diagnoses, potentially leading to better patient care and reduced unnecessary interventions.

Our work is particularly relevant in settings like Emergency departments, where timely and accurate diagnoses are crucial. We believe that our model can expedite the diagnostic process by providing relevant images promptly, potentially leading to improved patient outcomes and resource allocation.

The structure of the paper follows a logical sequence. We start by discussing the challenges posed by reduced diagnostic time and the uncertainty in complex cases. We then delve into the details of our novel architecture, explaining its design, strengths, and development process. Subsequently, we analyze detection accuracy by examining empirical results from our experiments. Finally, we provide perspectives on potential improvements and limitations, inviting further research and collaboration.

In conclusion, this work represents a step forward in intelligent image retrieval systems for medical imaging. Our deep learning-based model aims to aid radiologists in diagnosing lung and thoracic-related pathologies accurately, especially in challenging scenarios like Emergency departments.

## Related work

### CBMIR

Machine learning has emerged as a powerful tool for medical diagnosis, with deep learning showing particular promise. In radiology and cardiology, deep learning algorithms have been used to accurately detect abnormalities in medical images, revolutionizing the practice of medicine and improving patient outcomes ^5^. Deep learning algorithms have demonstrated high accuracy in image recognition tasks, which is particularly important in medical imaging, where detecting subtle patterns and abnormalities in images is crucial for accurate diagnosis ^6^.

Nearly in the past two decades, Content-Based Image Retrieval (CBMIR) has been a subject of extensive research the significant advent of large-scale databases, as noted by Wang ^5^. Several studies have made significant contributions to this field. For example, Tabatabaei ^6^ achieved an accuracy rate of 84% in CBMIR using the largest patch-annotated dataset in prostate cancer. Kalra ^7^ proposed Yottixel, a method for representing The Cancer Genome Atlas Whole Slide Images (TCGA WSIs) compactly to facilitate millions of high-accuracy searches with low storage requirements in real-time. Conversely, Mehta ^8^ proposed a CBMIR system for sub-images in high-resolution digital pathology images, utilizing scale-invariant feature extraction. Lowe ^9^ utilized Scale-Invariant Feature Transform (SIFT) to index sub-images and reported an 80% accuracy rate for the top 5 retrieved images. Lowe’s experiments were conducted on 50 ImmunohHistoChemistry (IHC) stained pathology images at eight different resolutions. Additionally, Hegde ^10^ used a manually annotated dataset pre-trained on a Deep Neural Network (DNN) to achieve top 5 scores for patch-based CBMIR at different magnification levels.

### BMIR: features representation

Machine learning, and specifically deep learning, has emerged as a powerful tool for medical diagnosis and has the potential to revolutionize the field of radiology ^7^. Deep learning algorithms can help radiologists to identify and interpret complex images, making their diagnoses more accurate and efficient. In the case of chest X-rays, deep learning algorithms can be trained to detect and classify abnormalities such as lung nodules and opacities, providing radiologists with an additional layer of analysis that can help to improve the speed and accuracy of their diagnoses ^8^. However, there has been a shortage of radiologists with the necessary expertise to meet the demand for these services ^9^. Convolutional Neural Networks (CNNs) have made remarkable progress in their ability to perform crucial medical applications at a level comparable to that of experts. This has had a profound impact on the field of medical imaging, enabling more accurate and efficient diagnoses. The use of CNNs in medical imaging has revolutionized the way radiologists approach diagnostic challenges, providing them with new tools and resources to improve their work. The widespread adoption of Convolutional Neural Networks (CNNs) in medical radiology and medical image analysis can be attributed to the advancements in computer vision techniques such as artificial intelligence and machine learning. The application of CNNs to triage patient abnormality from chest X-rays or CT scans has been widely researched, yielding valuable insights and advancements in the field. For example, ^7^ assessed the effectiveness of Conv Nets in detecting thoracic malignancies and demonstrated their potential to predict pulmonary findings and locate pathologies in medical images. Other works, such as Kundu et al. ^10^, focused on specific pathologies, using a custom CNN named DetNet59 ^11^ to detect pneumonia. These studies highlight the potential of CNNs to revolutionize medical imaging and improve diagnostic accuracy and speed. Features representation is a crucial aspect of Content-Based Medical Image Retrieval (CBMIR) systems and plays a key role in their ability to accurately identify and retrieve similar images. The features representation step in CBMIR involves finding a low-dimensional description of the image that captures its important characteristics and distinguishes it from other images. The use of CNNs in feature representation has enabled the development of CBMIR systems that can automatically present disease-matching similar images to the radiologist, reducing the time and effort required for manual image retrieval. This step is crucial for efficient retrieval in large databases and improves the accuracy of medical diagnoses by enabling the effective use of machine-learning techniques. The use of target detection algorithms has led to the development of two main types: two-stage and one-stage methods. Two-stage methods, like RCNN ^12^ use selective search to create sparse boxes and then classify and regress them with a CNN network. This method is known for high accuracy but can be slower and more complex. One-stage methods like YOLO and SSD, get the final detection result in one step through an end-to-end model. This approach is faster and simpler but may have lower accuracy ^13^. Jaeger et al. utilized a graph-cut approach to detect TB in CXR. They used a combination of object identification and content-based image retrieval techniques to locate and classify abnormalities ^14^. They made the databases public to aid in the early diagnosis of TB. Note that re-use and distribution of their work are strictly not permitted, except for open-access articles. ^15^, In their study, Ait Nasser and Akhloufi ^16^ implemented an ensemble learning approach that combined the predictions of three distinct deep convolutional neural network (DCNN) models—Xception, DenseNet-201^17^, and EfficientNet-B5—to classify chest X-ray (CXR) images into three categories: normal, lung disease, and heart disease. To train and evaluate the models, the authors compiled a dataset of 26,316 CXR images by aggregating data from both the VinDr-CXR ^18^ and CheXpert ^19^ datasets. Through their investigation, ^16^ demonstrated the potential of ensemble learning in leveraging the strengths of multiple deep learning models to improve the accuracy and generalizability of CXR image classification, which could have significant implications for the field of medical imaging. Cicero et al. ^20^ also demonstrated the potential of deep learning techniques in the analysis of medical images, highlighting the accuracy of DL models in detecting and classifying lung-related pathologies, which could have significant implications for improving clinical diagnosis and patient outcomes. Table 1 below is the comparison of different lung pathologies detection Techniques.

**Table 1:** Related work summary.

References	Method	Results
Souid et al.^7^	CNN used a Transfer learning strategy and wheighted loss to classify fourteen pathologies	Decent results in the pathology classification, achieving a 0.811 AUC score
Ait Nasser^16^	A similar approach to Souid et al.^21^ with some differentiation in the number of classes (from 8 to 14)	The method achieves very good results, the achieved AUC score is 0.949, however, the used dataset has a small number of samples which could limit the experimentation
Ayaz et al. ^22^	Ensemble of CNNs : Inception, InceptionResNet, VGG16, VGG19, MobileNet	The method Achieved an AUC of 0.934
Cicero et al.^20^	Using prived collected data from 2005 to 2015 consisting of 35,038 chest radiographs, the authors experimented with the Google LeNet5 CNN to construct an image classifier	The provided work has achieved an Aria Under Curve AUC of 0.964 for the classification of 5 pathologies and the beginning class. The score was obtained from testing over 2443 test samples
Rajaraman et al. ^23^	The presented work provides a new strategy to target Tuberculosis pathology, where they used a bone suppression strategy over multiple datasets	The obtained results were significantly improved compared to no-bone suppression applied with ResNet CNN The ResNet-BN achieved an AUC of 0.963 while the ResNet achieved only 0.89

## Methods

In this study, we aim to detect chest abnormalities through a two-step process. In the first stage, we utilize classic target detection approaches such as YOLOv5. Next, we employ an image classifier to perform binary classification to determine if an anomaly exists in the image. If the image is considered normal, we compare the normal probability to our set thresholds. If the probability falls below the low threshold, we keep the previous detection result. If it exceeds the high threshold, we disregard the YOLOv5 detection result and instead rely on the normal prediction. In all other cases, we add the normal prediction to the outcome.

### Pathologies detection

**Figure 2:**
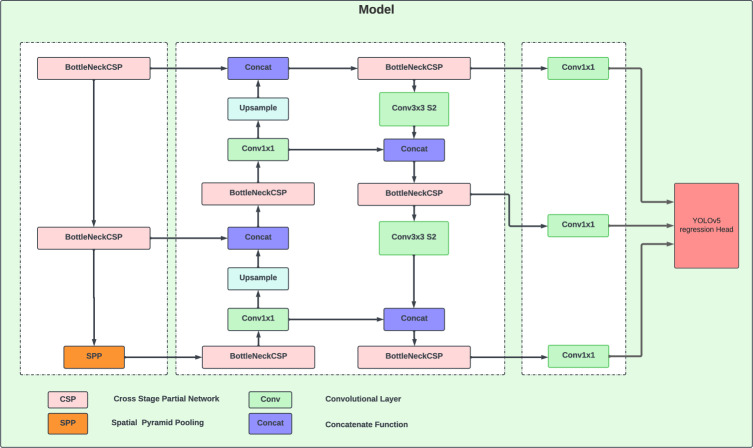
Simplified overview of the YOLOV5 main architecture.

In this study, we aim to detect lung-related pathologies in chest radiography scans. To do this, we employ a two-step procedure. First, we use object detection algorithms, such as YOLOv5, to identify the target. Then, we use an image classifier to perform two classifications (if there is an anomaly), and if the image is considered to be normal, we compare the normal probability to our thresholds. If the normal probability is below the low threshold, we maintain the previous detection result. If it exceeds the high threshold, we ignore the YOLOv5 detection result and use the normal prediction instead. If the normal probability falls in between, we add the normal prediction to the output ^24^. The use of chest radiography scans for detecting lung-related pathologies requires optimal features representation. There are two main types of algorithms for object detection using deep learning techniques: two-stage detection algorithms and single-stage detection algorithms. The former uses anchor boxes to obtain target location and classification results, while the latter, such as YOLO^14^, converts the object detection problem into regression by dividing the input image into grids and predicting the targeted objects. YOLOv5 is a recent and lightweight detection model that uses a CSPNet (Cross Stage Partial Network)^25^ structure to enhance features fusion and a Focus structure to reduce computation and speed up the processing. The network structure of YOLOv5 is shown in Fig. 2.

In what follows, we first present the overall framework of the model, and then we focus on the training phase and the improvements in detail.

### Image classification

**Figure 3:**
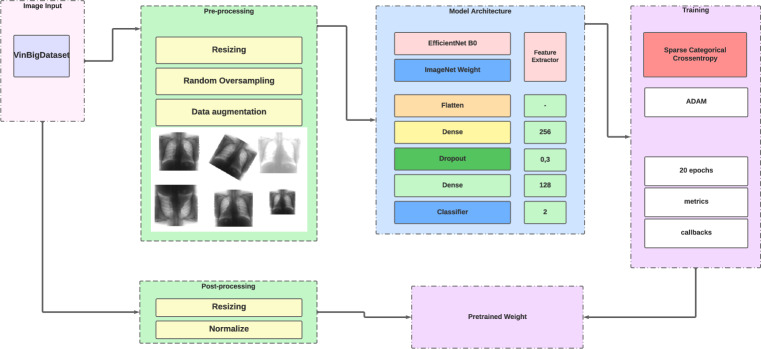
Used model architecture for abnormality triage based on Transfer Learning strategy.

EfficientNet is a family of efficient Convolutional Neural Networks (CNNs) designed to improve accuracy while reducing the number of parameters, computation, and memory usage required to perform image classification tasks. The main concept behind EfficientNet is to balance the scaling of network dimensions which leads to improved accuracy and low computational complexity. These models have been proven to achieve state-of-the-art performance on various benchmark datasets for image classification tasks, as well as being capable of adapting to other vision-related tasks ^26^**.** The presented work uses EfficientNetB0 ^26^ for the abnormality triage, also the transfer learning strategy is been used in this work, Figure 3 illustrates the architecture.

### Dataset processing

**Figure 4:**
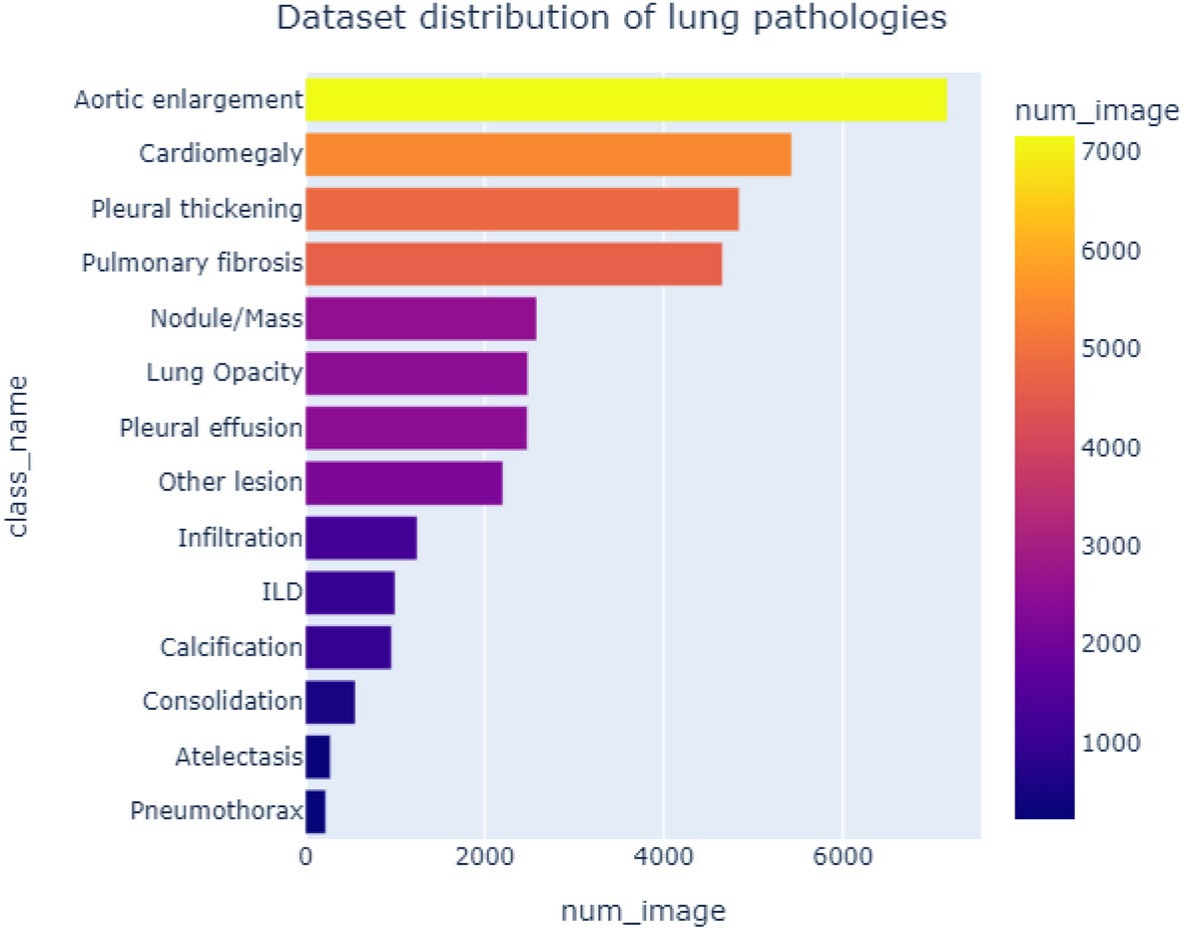
Pathologies distribution over the dataset: Originally the dataset has an added non-finding class, it’s being removed for better clarity of data visualization.

**Figure 5:**
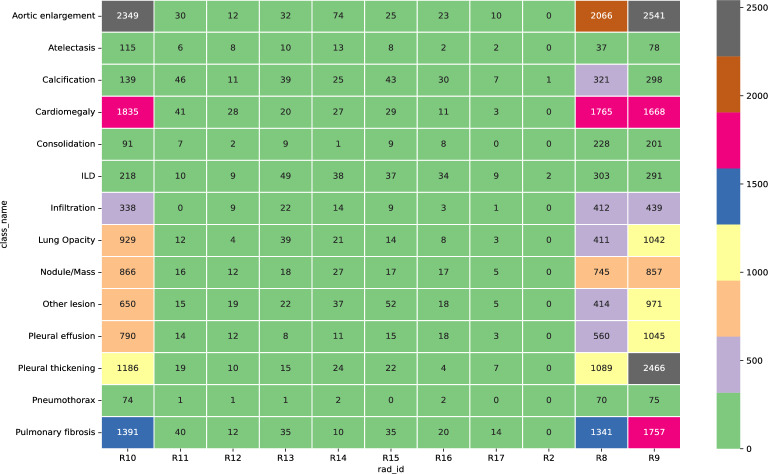
Radiologists’ pathologies annotations grid: most of the annotations are dispatched between the radiologist numbers 9, 8, and 10.

Our dataset was obtained from VinDr-CXR open-source dataset ^18^ for our analysis. Although the VinDr-CXR claims to contain annotations of various lung-related pathologies, including both global and local labeling, the open-source subset has a limited number of pathological categories. The VinDr-CXR uses the DICOM format to store chest X-ray images and de-identify the patient’s personal information. The dataset includes two sets of labels for each scan, the first annotation label identifies the presence of pathologies within each scan, while the second annotation localizes the specific pathology. The annotations were conducted by 18 radiologists, Figures 4 and 5 illustrate pathology sample distributions and the density of the annotations concerning the radiologists.

Based on the analysis of Fig. 5, it can be observed that a large portion of the radiologists' pathology annotations are concentrated among the 8th, 9th, and 10th radiologists, with the 10th radiologist having the highest number of annotations. This distribution suggests that there may be differences in expertise levels among radiologists.

The high number of annotations provided by the 10th radiologist may indicate a greater level of experience or specialization in the detection of pathologies. This information can be used to inform training and continuing education programs for radiologists to improve the accuracy and consistency of their pathology annotations.

**Figure 6:**
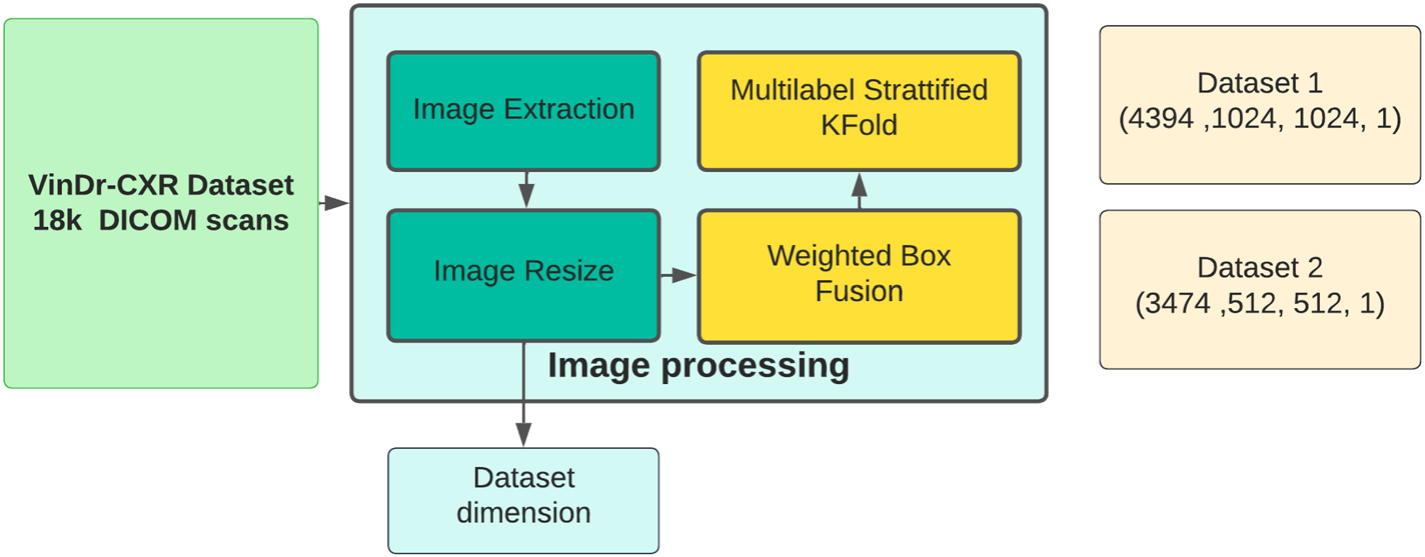
Data preprocessing pipeline.

However, the dataset faced the challenge of redundant local annotations and false annotations, which could negatively impact the performance of our model. To address this issue, we employed the Weighted Box Fusion (WBF) technique ^27^ techniques minimizes the redundant annotation and deleted false annotations, Figure 6 illustrates the data processing stage.

**Figure 7:**
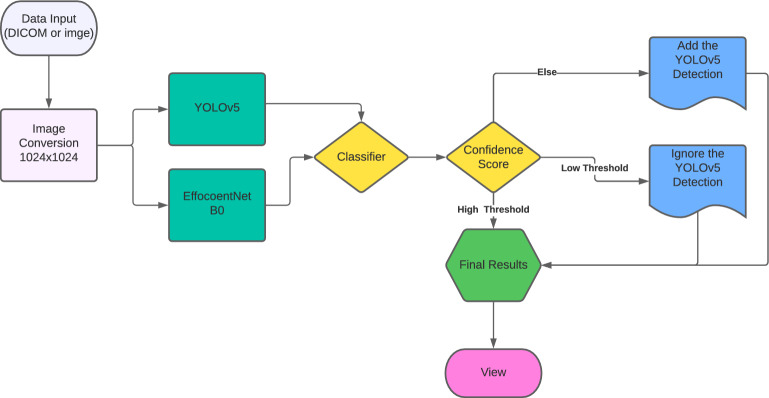
Features Representation workflow.

Originally The scan images were in the dimension of (2024, 2024, 1) which is very to process in the neural network, we converted the dataset to 2 datasets with dimensions of (512, 512, 1) and (1024, 1024, 1). The original dataset contains 7162 annotations, we notice a majority of aortic dissection-related symptoms composes the “other pathologies” class in the dataset, hence we included this class to produce more efficiency to the presented CAD solution, this class contains 2203 annotation from 1908 images. The WBF had minimized the annotations from 9365 to 4970 annotations, The model work is presented in the previous section and clearer in Fig. 7.

**Figure 8:**
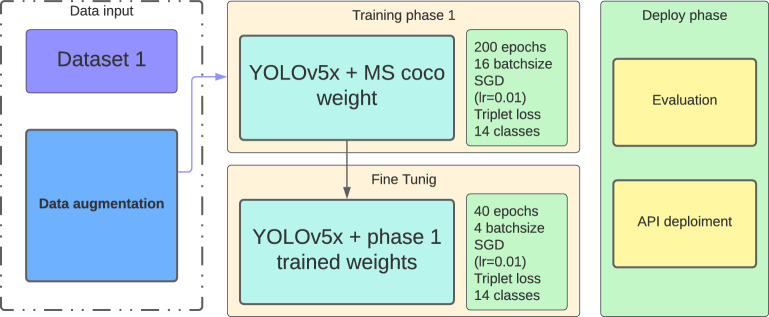
Proposed method two-staged training and evaluation pipelines.

The YOLOv5 is no doubt on the state-of-the-art object detection algorithms as mentioned in section A, however like any DL solution large datasets are a crucial requirement to build models, the presented dataset after cleaning and processing contains only 3474 scans which is far too small to build robust solution, hence our second contribution, this paper proposes two-staged training weights in order to achieve decent results. Figure 8 presents the training pipeline.

The presented training pipeline start with YOLOv5x algo with MS-coco dataset trained weights, this model trained on full-VinDr-CXR minus aortic enlargement and WBF with (1024, 1024, 1), as presented in the previous paragraph VinDr-CXR originally contains 15 classes. About the dataset split process, it was divided into 85% training and 15% validation. Data augmentation on the other hand focalize on implementing image blur, Median Blur, and CLAHE, which had been used an proven in the work of Chandra et al^28,29^, these data augmentation techniques are useful as data-generating processes. We used Stochastic Gradient Decent with a 0.01 initial learning rate as an optimizer to minimize the triplet loss combination (box loss, classification loss, and object loss). The model was trained for 200 epochs with batches of size 16 and 5 multilabel stratified KFold. Table 2 summarizes the model’s hyperparameters. The evaluation of the first model is not considered as a subject matter, as feedback from the first model, the coherence between pathologies creates major conflicts in the detection of the pathologies. The fine-tuning phase uses the obtained weight to train a new model. The same hyperparameters are used to train it, we use the dataset with 1024 dimensions for 40 epochs and batches with the size of 4. intention behind using the downsized dataset (1024, 1024, 1) is to maximize features representation as most medical images u high resolutions images model inference during deployment. We obtain very decent results during evaluations; these experiments were executed using a rented cloud instance with a single CPU and Nvidia A100 graphic processor, and the code was written in python.

**Table 2:** Models hyperparameters settings.

Parameters	Stage 1	Stage 2	Classifier
Weight decay	0.0005	0.0005	–
Batch size	16	4	16
Learning rate	0.01	0.01	0.01
epochs	200	40	20

As for the abnormality trail training, it uses the same dataset for the previous section, but it gets a different processing pipeline, the dataset labels were regrouped into two classes abnormal or no finding class. The model then was trained using smaller images specifically with sizes (224,224, 3) which is the typical data size to apply transfer learning. The model was trained during 20 epochs, with 16 batch sizes, Adam optimizer with binary cross entropy loss. The model also uses two major blocks, the features extraction block is the EfficientNetB0 with imageNet weights, and the classifier block is composed of Flatten layer, two dense layers with the sizes of 256 and 128 respectively, and a classifier layer with two element vectors activated with sigmoid function. The model achieves stable results which will be presented later in this work.

In our models, the initial range of the hyperparameters is shown in Table 2 and sequentially optimized as the model trains.

### Model evaluation

This section describes some evaluation metrics used in our experiment. Common metrics for measuring the performance of detection and classification algorithms include determining model precision, sensitivity, and f1-score, also to better evaluate the object detection model it is recommended to calculate mean average precision (mAP). Other metrics (classifiers) are very popular and do not need to be presented. Model precision presents the quality, their formula is illustrated as follows:

1 $$\mathrm{Precision}=\frac{\mathrm{TP}}{\mathrm{TP}+\mathrm{FP}}$$

The sensitivity formula is:

2 $$\mathrm{Sensitivity}=\frac{\mathrm{TP}}{\mathrm{TP}+\mathrm{FN}}$$

The sensitivity metric represents the quantity of the accurately detected samples, where both in (1) and (2), TP refers to the True positive, FP represents the False positive, and FN refers to False Negative.

The F1-score formula is:

3 $$F_{{1 - {\text{score}}}} = 2 \times \frac{{{\text{precision}} \times {\text{recall}}}}{{{\text{precision}} + {\text{recall}}}}$$

Finally, the mean avg precision (mAP) is the required metrics to measure object detection performance by summarizing the precision-sensitivity curve into a single value representing the avg of all precisions, it is calculated as follows:

4 $$mAP = \frac{1}{{|{\text{classes}}|}}\sum\limits_{{c \in {\text{classes}}}} {\frac{{|{\text{TP}}_{c} |}}{{|{\text{TP}}_{c} | + |{\text{FP}}_{c} |}}}$$

## Experimental result

### Results evaluation

**Figure 9:**
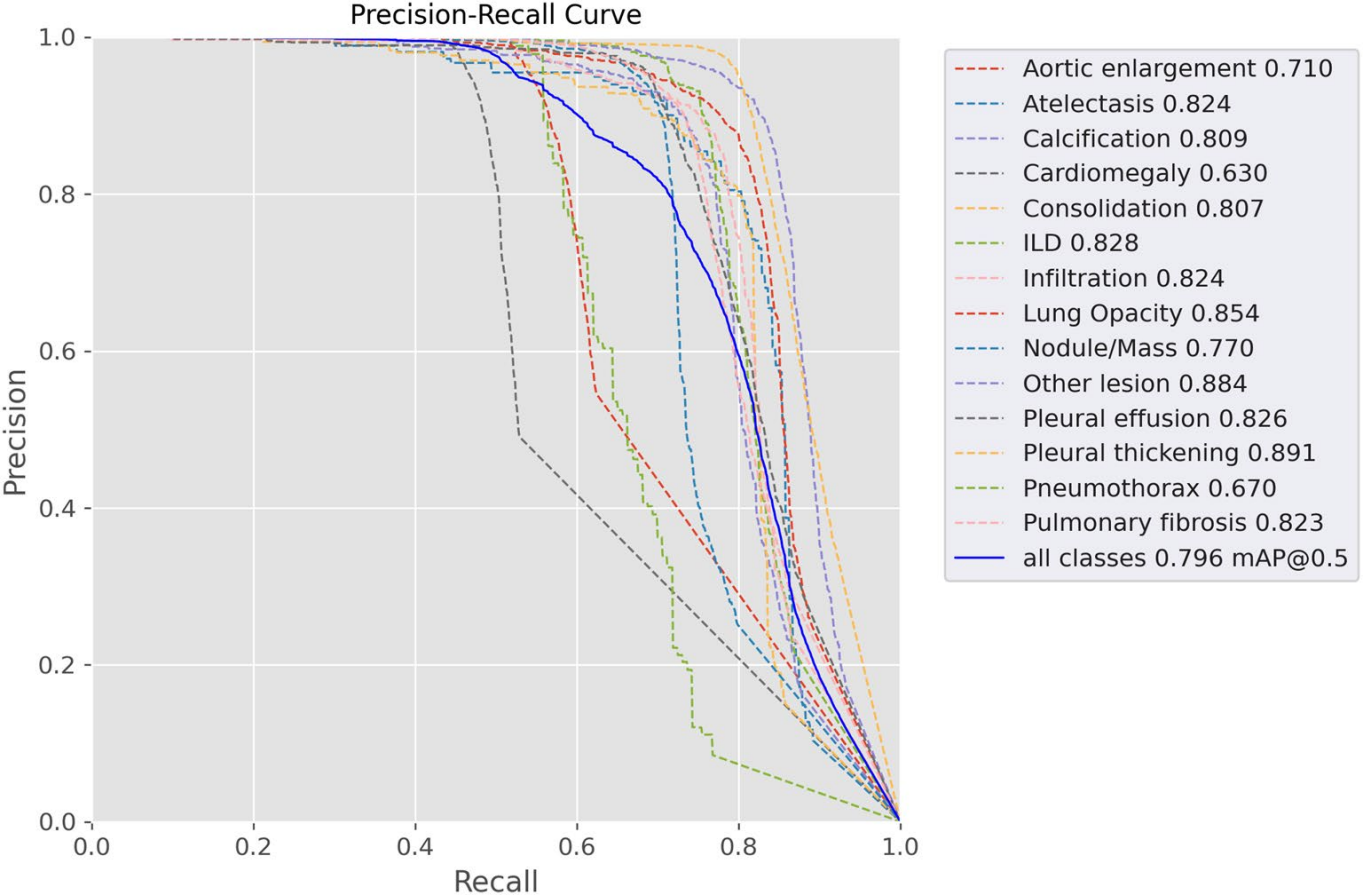
Proposed method validation of mean average precision**;** the majority of the pathologies 386 have smooth curves indicating the capability of the model in detecting these pathologies.

Prior to this section, we presented the lung pathologies detection model, we also detailed the used dataset processing and the used metrics to evaluate the gained models, we used mAP with a threshold of 0.5 and mAP with a threshold between [0.5:0.95]. We achieved a maxim F1-score of 0.792 at a threshold of 0.162, the average precision of all pathologies achieved a maximum of 0.909, and the model also achieves an average sensitivity of 0.796, Table 3 summarizes the achieved results, when discussing the result of the aortic dilatation prediction result separately, the precision of detecting Cardiomegaly achieves 0.991, respectively sensitivity achieve 0.44, the average mAP with threshold of 0.5 score achieves a maximum of 0.63, the most stable pathology is the Pleural Thickening with the precision of 0.941, sensitivity of 0.804 and mAP with threshold of 0.5 of 0.891. The hardest pathology to detect is Pneumothorax due to the dataset amount. The model shows a fairly decent result, compared to the literature works. This result also reflects the role of the other pathology class in maximizing the detection accuracy, the Fig. 9 illustrated the precision-sensitivity curve maximum of 0.0606, the most stable pathology is the Pleural Thickening with a precision of 0.941, sensitivity of 0.804, and mAP with threshold of 0.5 of 0.891. The hardest pathology to detect is Pneumothorax due to the dataset repartition. The model shows a fairly decent result, compared to the literature works. This result also reflects the role of the other pathology class in maximizing the detection accuracy, the Fig. 9 illustrated the precision-sensitivity curve.

**Table 3:** Models hyperparameters results including Precision, Sensitivity, F1-score and mAP.

Pathologies	Precision	Sensitivity	F1-score	mAP 0.5	mAP [0.5:0.95]
Aortic enlargement	0.985	0.521	0.681	0.71	0.559
Atelectasis	0.833	0.778	0.804	0.824	0.593
Calcification	0.815	0.769	0.791	0.809	0.587
Cardiomegaly	0.991	0.44	0.609	0.63	0.487
Consolidation	0.875	0.74	0.801	0.807	0.607
ILD	0.949	0.716	0.816	0.828	0.628
Infiltration	0.912	0.739	0.816	0.824	0.619
Lung Opacity	0.894	0.781	0.833	0.854	0.643
Nodule/Mass	0.904	0.701	0.789	0.77	0.555
Other lesions	0.897	0.831	0.862	0.884	0.652
Pleural effusion	0.887	0.72	0.794	0.826	0.567
Pleural thickening	0.941	0.804	0.867	0.891	0.631
Pneumothorax	0.86	0.567	0.683	0.67	0.521
Pulmonary fibrosis	0.91	0.728	0.808	0.823	0.599
avg	0.909	0.702	0.792	0.796	0.589

**Figure 10:**
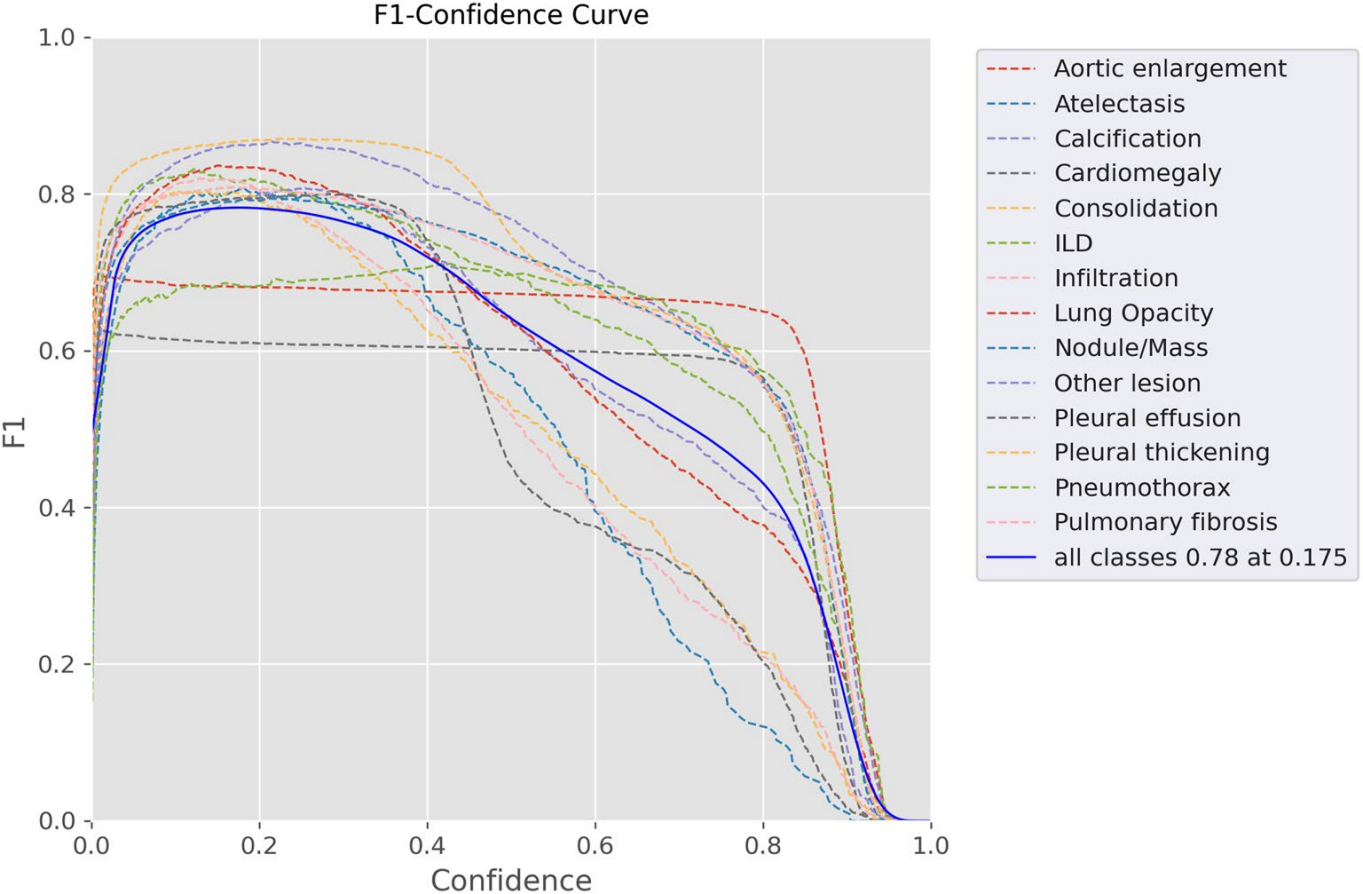
Proposed method validation of F1-score**.**

The performance of a detection model was evaluated using various evaluation metrics. The analysis revealed a maximum F1-score of 0.792 at a threshold of 0.175, as depicted in Fig. 10. Furthermore, the accuracy of the single model was assessed using the mean average precision (mAP) metric, which yielded a value of 78%, as illustrated in Fig. 10. Overall, the findings of this work suggest that the suggested model and combined model offer improved performance compared to the baseline model, as evidenced by the improvement in F1-score and mAP metrics. These results suggest that the proposed approach holds promise for the detection task.

The EfficientNetB0 classifier model demonstrated robust performance, as described in the training section. The model achieved a positive predictive value (PPV) of 0.935, a negative predictive value (NPV) of 0.977, a specificity of 0.842, for the abnormal class, and a prevalence of 0.70 on the separate test data set. Furthermore, when evaluated using classical classification metrics on a sample size of over 3000 images, the model achieved a mean performance score of 93%, Table 4 illustrates the obtained results.

**Table 4:** the abnormality triage model results: the binary classification model that detect normal and abnormal simples.

Metrics	Normal	Abnormal
Accuracy	0.937333	0.937333
F1-score	0.889542	0.956259
Negative predictive value	0.935792	0.941542
Positive predictive value	0.941542	0.935792
Precision	0.941542	0.935792
Sensitivity	0.842984	0.977640
Specificity	0.977640	0.842984

**Figure 11:**
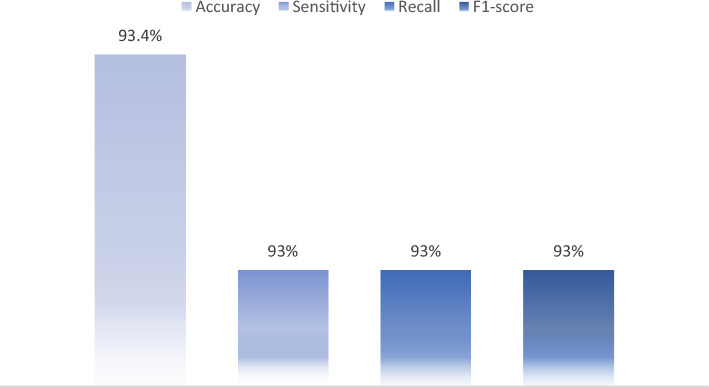
Classic classification metrics for the abnormality trial model.

These results are further supported by the values presented in Fig. 11, which displays the classical classification report. Overall, the findings suggest that the EfficientNetB0 classifier model offers strong predictive power and could be a valuable tool for medical image analysis.

The proposed method successfully achieves the primary aims of the study, specifically the successful triage of abnormalities and a reduction in false positive rates. Although a high false positive rate may not be a severe issue in medical image analysis, it is still crucial to minimize it as much as possible to avoid expending time and resources on unnecessary interventions or follow-ups. As such, the reduction of false positives resulting from this approach offers a significant advantage in enhancing the precision and effectiveness of medical image analysis.

Additionally, the second model presented in this study also demonstrates promising results when compared with related architectures and objectives. By combining these two models, the proposed architecture offers more stable performance, as the detector model can accurately distinguish between non-finding (normal) patient cases. Overall, this approach offers a comprehensive and effective solution to triage abnormalities and reduce false positive rates in medical image analysis.

## Discussion

The outcomes of our study have been thoroughly examined, aiming to offer a comprehensive interpretation and analysis of the results obtained. This section presents an in-depth discussion of the significance of our findings, supported by meaningful insights and explanations.

Our study successfully achieves its primary objectives by effectively detecting various lung-related pathologies, such as pneumothorax, alongside cardiac-related conditions like cardiomegaly and thoracic aortic enlargement. This proactive approach holds the potential to mitigate potential acute and chronic pathologies, demonstrating the clinical relevance of our work.

Importantly, our findings make significant contributions to the existing body of knowledge within this domain. Notably, our method yields competitive results that challenge established state-of-the-art approaches. Through careful consideration of weakly supervised detection-based strategies, we effectively navigate the complexities of our target task.

Comparing our results with the prior literature reveals intriguing insights. Luo et al.'s ^30^ work showcases notable similarities in architectural choices, leading to commendable chest X-ray reporting results. Interestingly, we surpass their achievements, particularly evident in the case of ResNet50 + YOLOv5, where our approach achieves an impressive mean average precision (mAP) of 0.546 at an IoU threshold of 0.95. This represents a substantial enhancement compared to their reported mAP of 0.254. Furthermore, even in comparison to YOLOv5's standalone performance with a mAP of 0.244, our method continues to shine.

Our method's performance against Pham et al.'s ^31^ work echoes a similar trend. Their approach, employing a similar architecture, reaches a mAP of 0.466 at a threshold of 0.95. Strikingly, our model excels by achieving a 14% improvement, reinforcing the robustness and efficacy of our proposed solution.

**Figure 12:**
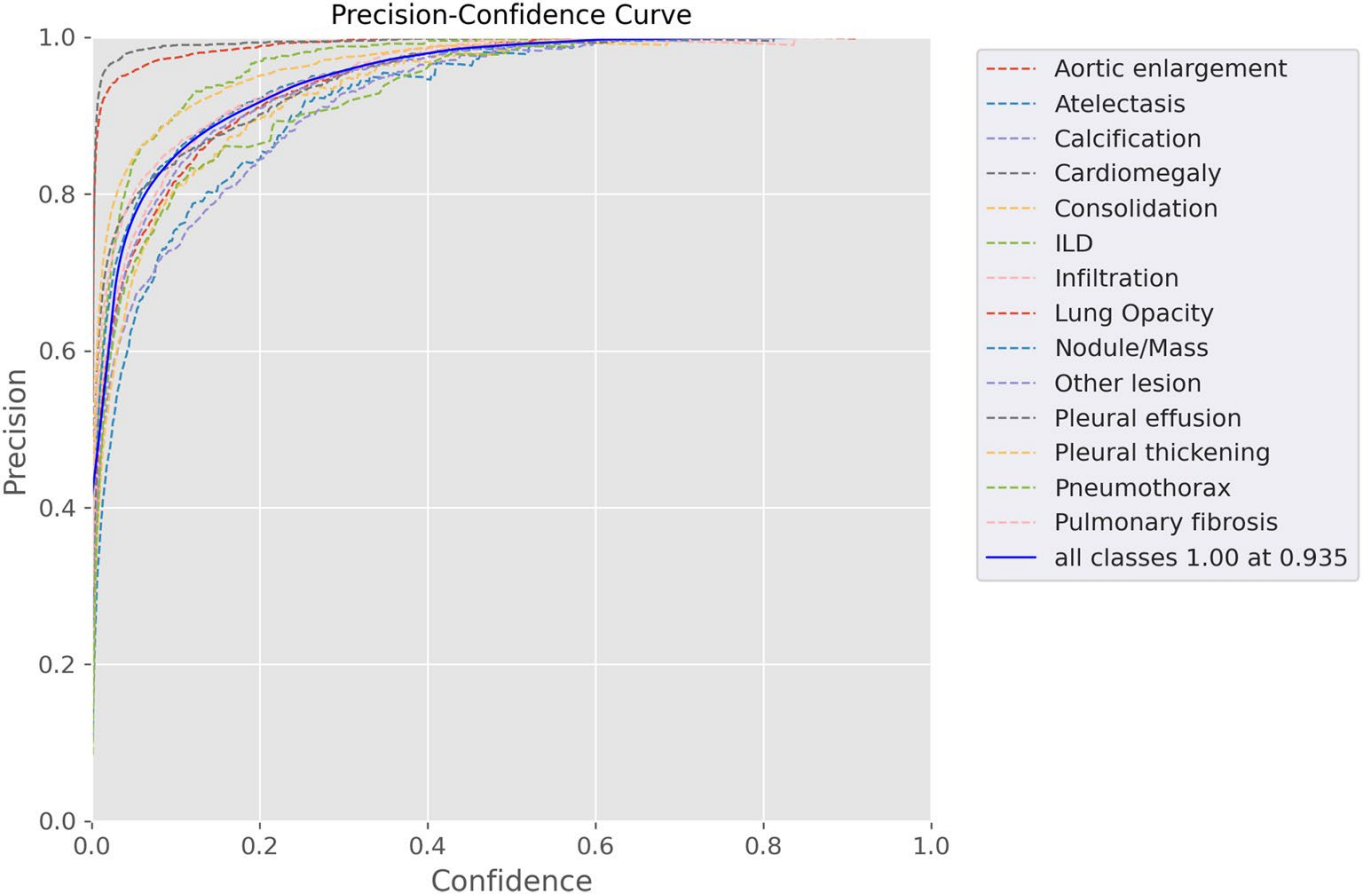
Proposed method validation of precision.

Figure 12 provides an insightful depiction of our precision (sensitivity) results, offering a clearer understanding of the achieved sensitivity outcomes.

**Figure 13:**
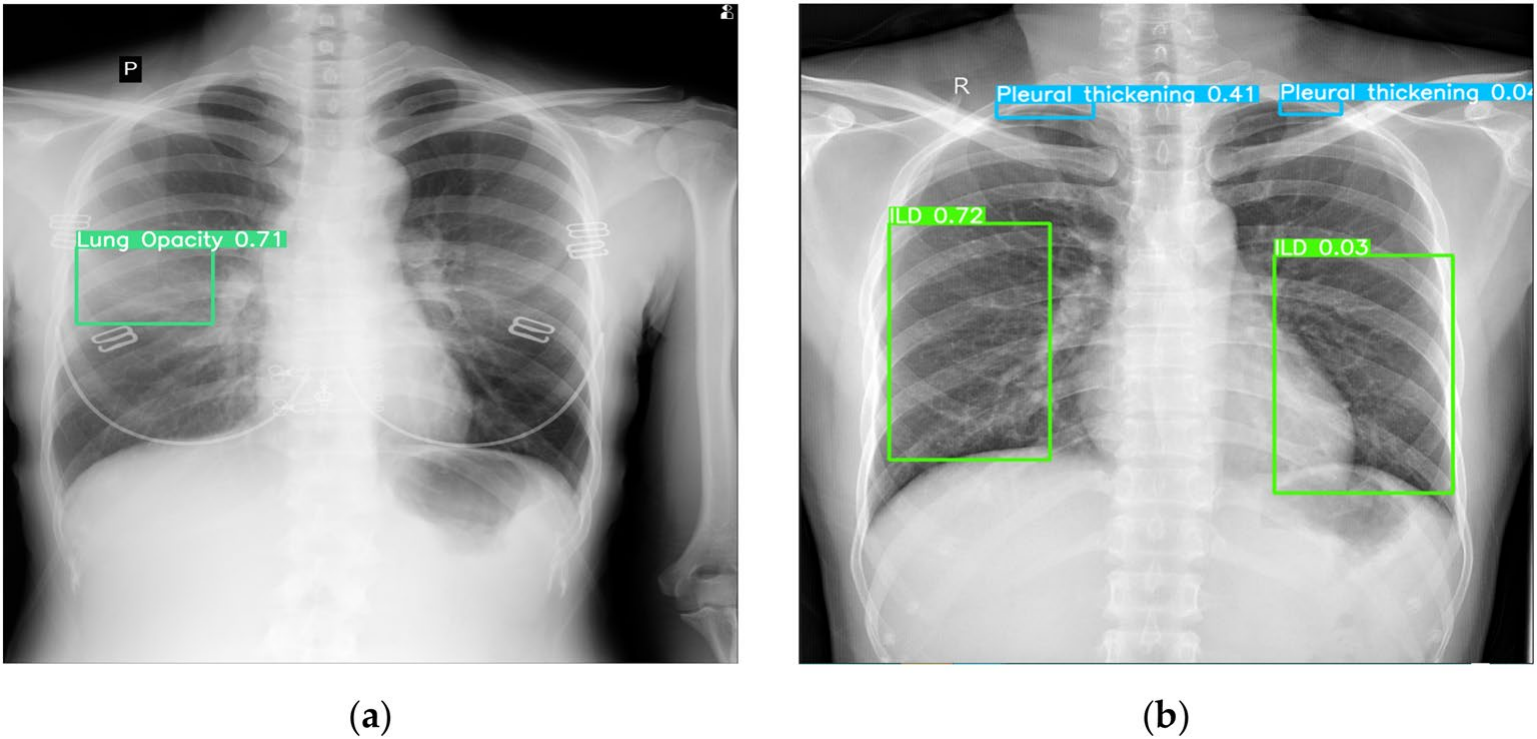
Prediction samples from the deployed model: (**a**) Prediction of the patient scan showing the presence of Lung opacity; (**b**) Prediction from another chest radiograph exam that shows the existence of four findings and pathologies: Interstitial lung disease (ILD), Pleural thickening.

Some works provide specific pathologies detection such as the work of Luo et al. ^30^, also the work of Nguyen et al. ^32^ implement detection solutions, the aortic enlargement mAP of 0.531, and the scan presented in Fig. 13 shows some predictions. The result provided by Luo et al. ^30^ are presented with a threshold of 0.6, nevertheless our model still remarkably surpass these results. Other related works were limited to TAD prediction without localizing it in a scan, please check Table 5.

**Table 5:** Proposed model’s performance compared to the prior research.

Methods	mAP 0.5	mAP [0.5:0.95]
^30^: YOLOv5 + ResNet50	–	0.254
^30^: YOLOv5	–	0.244
^30^: Fast RCNN	–	0.234
^30^: EfficientDet		0.231
^31^: Faster RCNN	0.417	–
^31^: YOLOv5 + ResNet50	0.812	0.466
Ours: YOLOv5 + EfficientNet	0.884	0.546

It's worth noting that we acknowledge the limitation in our F1 scores presented in Table 3. We are cognizant that these scores may not appear as promising as desired. We attribute these results to the inherent limitations of the dataset used, specifically data imbalances, particularly in the annotations. Addressing these imbalances in chest X-ray annotations is a challenge due to the coupled nature of these annotations. Despite these challenges, our study strives to contribute within the context of these constraints, offering insights into the performance of our approach.

To ensure good performance, modern machine learning models typically require large amounts of quality annotated data. Meanwhile, the data collection and annotation processes are usually performed manually and consume a lot of time and resources and settings. It is often not feasible to obtain sufficient training data. Currently, data augmentation is the most effective way of alleviating this problem. The main goal of data augmentation is to increase the volume, quality and diversity of training data. In the next work we focus is on more recent and advanced data augmentation techniques to solve this issue.

## Conclusions

In this paper, we have presented a novel approach for the automatic detection of chest abnormalities in X-ray images by combining the YOLOv5 object detection technique with the EfficientNetB0 binary CNN classifier. Our results demonstrate that this approach is highly effective and has the potential to significantly improve the accuracy and efficiency of medical image analysis.

Furthermore, our approach has important implications for the development of content-based medical image retrieval systems. By leveraging the feature representation capabilities of deep learning models such as YOLOv5 and EfficientNetB0, it is possible to develop an image retrieval system that enables healthcare professionals to search for and access relevant medical images with greater ease and accuracy.

Overall, our findings highlight the importance of using advanced deep-learning techniques and data preparation methods to improve medical image analysis. We believe that our approach represents a significant contribution to the field and has the potential to provide better healthcare solutions for patients.

While our current study marks a significant advancement in the domain of chest abnormality detection, several avenues remain unexplored. One promising avenue is the integration of multi-modal data, combining information from diverse medical imaging sources to further refine the accuracy and robustness of our approach. Additionally, exploring transfer learning strategies could facilitate the adaptation of our model to diverse medical specialties, expanding its applicability across various diagnostic tasks. Furthermore, the incorporation of explainable AI techniques could enhance the transparency of our model's decision-making process, fostering greater trust among healthcare practitioners. Finally, addressing the challenges posed by limited annotated data through innovative data augmentation methods or semi-supervised learning approaches holds the potential to bolster our model's generalization capabilities. By delving into these directions, future research can continue to advance the efficacy and versatility of automated medical image analysis systems.

## Data availability

The datasets used during the current study are available from the corresponding author on reasonable request.
